# Evaluating the optimal timing of surgical antimicrobial prophylaxis: study protocol for a randomized controlled trial

**DOI:** 10.1186/1745-6215-15-188

**Published:** 2014-05-24

**Authors:** Edin Mujagic, Tibor Zwimpfer, Walter R Marti, Marcel Zwahlen, Henry Hoffmann, Christoph Kindler, Christoph Fux, Heidi Misteli, Lukas Iselin, Andrea Kopp Lugli, Christian A Nebiker, Urs von Holzen, Fabrizio Vinzens, Marco von Strauss, Stefan Reck, Marko Kraljević, Andreas F Widmer, Daniel Oertli, Rachel Rosenthal, Walter P Weber

**Affiliations:** 1 Department of General Surgery, University Hospital of Basel, Spitalstrasse 21, 4031 Basel, Switzerland; 2 Department of General Surgery, Hospital of Aarau, Tellstrasse, 5000 Aarau, Switzerland; 3 Institute of Social and Preventive Medicine, University of Bern, Finkenhubelweg 11, 3012 Bern, Switzerland; 4 Department of Anesthesiology, Hospital of Aarau, Tellstrasse 15, 5001 Aarau, Switzerland; 5 Department of Infectious Diseases, Hospital of Aarau, Tellstrasse 15, 5001 Aarau, Switzerland; 6 Department of Anesthesiology, University Hospital Basel, Spitalstrasse 21, 4031 Basel, Switzerland; 7 Department of Infectious Diseases, University Hospital Basel, Spitalstrasse 21, 4031 Basel, Switzerland

**Keywords:** Randomized controlled trial, Surgical antimicrobial prophylaxis, Surgical site infection

## Abstract

**Background:**

Surgical site infections are the most common hospital-acquired infections among surgical patients. The administration of surgical antimicrobial prophylaxis reduces the risk of surgical site infections . The optimal timing of this procedure is still a matter of debate. While most studies suggest that it should be given as close to the incision time as possible, others conclude that this may be too late for optimal prevention of surgical site infections. A large observational study suggests that surgical antimicrobial prophylaxis should be administered 74 to 30 minutes before surgery. The aim of this article is to report the design and protocol of a randomized controlled trial investigating the optimal timing of surgical antimicrobial prophylaxis.

**Methods/Design:**

In this bi-center randomized controlled trial conducted at two tertiary referral centers in Switzerland, we plan to include 5,000 patients undergoing general, oncologic, vascular and orthopedic trauma procedures. Patients are randomized in a 1:1 ratio into two groups: one receiving surgical antimicrobial prophylaxis in the anesthesia room (75 to 30 minutes before incision) and the other receiving surgical antimicrobial prophylaxis in the operating room (less than 30 minutes before incision). We expect a significantly lower rate of surgical site infections with surgical antimicrobial prophylaxis administered more than 30 minutes before the scheduled incision. The primary outcome is the occurrence of surgical site infections during a 30-day follow-up period (one year with an implant in place). When assuming a 5% surgical site infection risk with administration of surgical antimicrobial prophylaxis in the operating room, the planned sample size has an 80% power to detect a relative risk reduction for surgical site infections of 33% when administering surgical antimicrobial prophylaxis in the anesthesia room (with a two-sided type I error of 5%). We expect the study to be completed within three years.

**Discussion:**

The results of this randomized controlled trial will have an important impact on current international guidelines for infection control strategies in the hospital. Moreover, the results of this randomized controlled trial are of significant interest for patient safety and healthcare economics.

**Trial registration:**

This trial is registered on ClinicalTrials.gov under the identifier NCT01790529.

## Background

### The importance of surgical research for the prevention of surgical site infections

Surgical site infections (SSI) account for 14% to 16% of all nosocomial infections in hospitalized patients and are considered the most common form of nosocomial infection among surgical patients [1]. Despite a variety of different prevention measures, as many as 5% of all patients undergoing surgery develop SSI, which lead to additional morbidity and mortality [2-4]. Patients with SSI require a longer hospital stay, more nursing care, and often readmissions with additional surgery [5-8]. The combined additional direct and indirect costs of treating SSI are substantial [9-12]. Hospitals are under pressure to reduce costs, and efforts to decrease the rate of SSI have therefore become a matter of increasing interest for surgeons, operating room nurses, anesthesiologists, infection control professionals and healthcare epidemiologists [13]. Nowadays, SSI are considered to reflect the quality of care in a hospital, as they are potentially preventable complications directly linked to surgery. However, many of the current recommendations of the Centers for Disease Control and Prevention (CDC) and the World Health Organization are based on evidence from observational studies in the absence of confirmatory trials [1,14].

### Surgical antimicrobial prophylaxis

The introduction of routine surgical antimicrobial prophylaxis (SAP) was a breakthrough in the prevention of SSI [15]. Today, SAP is administered in surgical units on a daily basis. Based on evidence from observational and randomized controlled trials (RCT), there is widespread agreement for the use of SAP before all gastrointestinal, oropharyngeal and gynecological procedures [15-23]. There is ongoing controversy about the use of SAP for ‘clean’ operations, in which the absolute number of infections is low and the number of administrations of SAP needed to prevent one infection is high. It is well accepted for the following clean surgeries, in which the consequence of any infection is severe: orthopedic prosthesis placement, vascular surgery, open-heart surgery and neurosurgery procedures [24-33]. A reduction in infection rates is well documented for other clean operations such as breast, varicose vein and herniorrhaphy procedures [34-38]. However, in these procedures the morbidity of the infection is generally low and the benefits of SAP must be balanced against its costs and possible adverse effects.

Several antibiotics have been shown to reduce the incidence of SSI. Many hospitals, especially in the US, use very complex SAP regimes with a variety of antimicrobial drugs that have different pharmacokinetics depending on the type of surgery performed [15,16,39,40]. Current guidelines, however, suggest that single-shot administration of a first-or second-generation cephalosporin is sufficient for optimal prevention of SSI in the absence of high rates of resistant bacteria [41]. Due to a limited anaerobic activity of most cephalosporins, treatment is supplemented with metronidazole where indicated. The time interval to motivate redosing is generally set at four hours. Therefore, in several hospitals in Switzerland, including the University Hospital of Basel and the Hospital of Aarau, SAP consists of single-shot administration of cefuroxime (a second-generation cephalosporin), combined with metronidazole in colorectal and proctologic surgery, that is repeated in operations exceeding four hours.

### When to administer surgical antimicrobial prophylaxis?

Before the late 1960s, most prophylactic antibiotics were administered after the end of a surgical procedure and were found to be ineffective [42]. In 1961, Burke [43] showed the timing of SAP to be crucial in animals. Subsequent studies in humans suggested that adequate tissue levels of an appropriate antibiotic during surgery were essential [21,44-47]. The observational landmark study by Classen and colleagues [48] in 1992 provided the basis for the antimicrobial agents to be administered within two hours before skin incision. Other authors narrowed the optimal window for SAP to less than 60 minutes before skin incision [49,50]. Importantly, two large prospective studies observed the lowest risk of SSI when SAP was given within 30 minutes prior to incision, and the National Surgical Infection Prevention Project simply recommends administering SAP as close to the incision time as possible [51-53]. Similar statements are made in European guidelines [54,55].

However, despite the obvious importance of infection control by SAP, none of the recommendations on the optimal timing is backed by evidence obtained in an RCT. The historic study by Classen and colleagues [48] was conducted when it was standard practice to administer antibiotics to all patients for at least 24 hours, which was extended to ≥48 hours in more than 80% of cases. Moreover, the antibiotics administered had widely varying half-lives. Consequently, the Classen *et al*. time window may not be appropriate for an optimal prevention of SSI as practiced today with single-shot regimes. In addition, there is little evidence in the literature to suggest that tissue levels of cefuroxime could reach the minimum inhibitory concentration within a few minutes after administration. Different authors have attained appropriate tissue levels of cefuroxime anywhere from 20 to 90 minutes after intravenous application [56-58]. The translocation of skin microorganisms into the wound during incision is the first vulnerable phase of surgery, and administering SAP only a few minutes before incision might not be optimal to achieve the tissue levels required to prevent SSI.

Several recent studies of other antimicrobial agents with different pharmacokinetics support this hypothesis. *In vivo* microdialysis, for example, is a new development that allows the measurement of continuous unbound antibiotic concentrations in muscular and subcutaneous interstitial fluid during surgery. Hutschala *et al*. [59] described this *in vivo* approach of microdialysis to measure continuous tissue levels of cefazolin. Importantly, the authors state that ‘Cefazolin should be administered at least 60 minutes before skin incision to guarantee for optimal tissue concentration at the beginning of surgery. Vast inter-individual differences were observed for the time required to reach maximum interstitial concentrations. So it seems reasonable to administer the prophylactic antibiotic as early as possible before skin incision’ [59].

Two recent prospective observational studies of other antimicrobial agents with different pharmacokinetics are noteworthy [60,61]. In one, the administration of vancomycin 16 to 60 minutes before incision in coronary artery bypass surgery was associated with the lowest risk of SSI [60]. The other study showed that the rate of SSI after uncomplicated open appendectomy is lower when the antibiotic is administered more than one hour versus one hour or less before surgery [61]. Finally, the results of an observational cohort study performed at the University Hospital of Basel suggest that the ideal timing of SAP is between 74 and 30 minutes before skin incision [62].

### Relevant ongoing research

As of 27 January 2014, there were 198 studies on ‘surgical site infection AND prevention’ or ‘surgical site infection AND prophylaxis’ - excluding the present one - registered on ClinicalTrials.gov*,* 88 of which were open. There were 122 studies found using the terms ‘surgical site infection AND antibiotic’, all of which were covered by the above search criteria. Finally, there were 75 studies found using ‘surgical site infection AND prophylaxis AND antibiotic’.

None of these 198 studies investigates the incidence of SSI as a function of SAP timing. Instead, most of them assess the impact of multiple SSI prevention measures on the risk of SSI, such as nasal decontamination, surgical hand antisepsis, preoperative patients’ skin cleansing, hair clipping, supplemental oxygen, local warming and antibacterial sutures. Some studies focus on different aspects of SAP, such as the overall efficacy of different types, doses, durations or ways of applications of SAP in specific subsets of patients, others assess the impact of quality improvement programs on compliance with current guidelines.

In summary, SSI are frequent surgical complications that have an important impact on healthcare costs. SAP prevents SSI and has therefore become a main stem of surgical infection control in many surgical interventions. Current guidelines for the correct timing of SAP, however, are still based on observational and pharmacokinetic studies. Such studies have recently achieved discordant results. A well-conducted RCT seems warranted to obtain a clear answer on the optimal timing. There is currently no ongoing or planned trial registered on ClinicalTrials.gov to address this question. The use of a single-shot single-drug SAP regime at two tertiary referral centers in Switzerland provides an ideal setting to plan and conduct an RCT on the optimal timing of SAP.

### Current state of own research in the field

In a quality assessment study conducted at the University Hospital of Basel, we prospectively followed 6,283 consecutive general, oncologic, vascular and orthopedic trauma surgery procedures closely for evidence of SSI, and then analyzed the dataset for the influence of various SSI risk factors. The *a priori* hypothesis of this study was that the timing of SAP had a significant impact on the occurrence of SSI. The lowest rates of SSI were observed with the antibiotics being administered between 74 and 30 minutes before surgery, and the association remained virtually unchanged when controlling for patient and procedural risk factors for SSI [62].

Within that cohort study, a matched case-control study was conducted on the economic impact of in-hospital SSI at the University Hospital of Basel. The mean additional hospital cost per SSI was 19,638 CHF (95% confidence interval 8,492 to 30,784 l) or 12,765 Euro (95% confidence interval 5,520 to 20,010) [63]. Further analyses of this cohort study suggest that glove perforation is associated with an increased SSI risk in the absence of SAP, but show no statistically significant associations between transfusion, anemia or tutorial assistance and the risk of SSI [64-66]. A review of the microbiological features of SSI in this series demonstrates the absence of multiresistant germs and validate the continuous use of single-shot single-drug SAP with cefuroxime (plus metronidazole in colorectal and proctologic surgery) [67]. Finally, a secondary analysis has been conducted to assess the sensitivity of our clinicians SSI surveillance system to register in-hospital SSI [68].

### Aims

The final goal of this present project is to reduce the rate of SSI by providing level I evidence on the optimal timing of the administration of SAP in general, oncologic, vascular and orthopedic trauma surgery. We expect that evidence to influence international guidelines for SAP. The hypothesis to be tested is that the risk of SSI is significantly lower with cefuroxime (plus metronidazole in colorectal surgery) applied in the anesthesia room (75 to 30 minutes before surgery) as compared to its administration in the operating room (within the final 30 minutes before surgery) [62].

## Methods/Design

### Study design and sites

This bi-center prospective RCT is being conducted at the University Hospital of Basel and the Hospital of Aarau, two tertiary referral centers in Switzerland. The trial has been registered on ClinicalTrials.gov under the identifier NCT01790529*.*

### Patients

**Figure 1:**
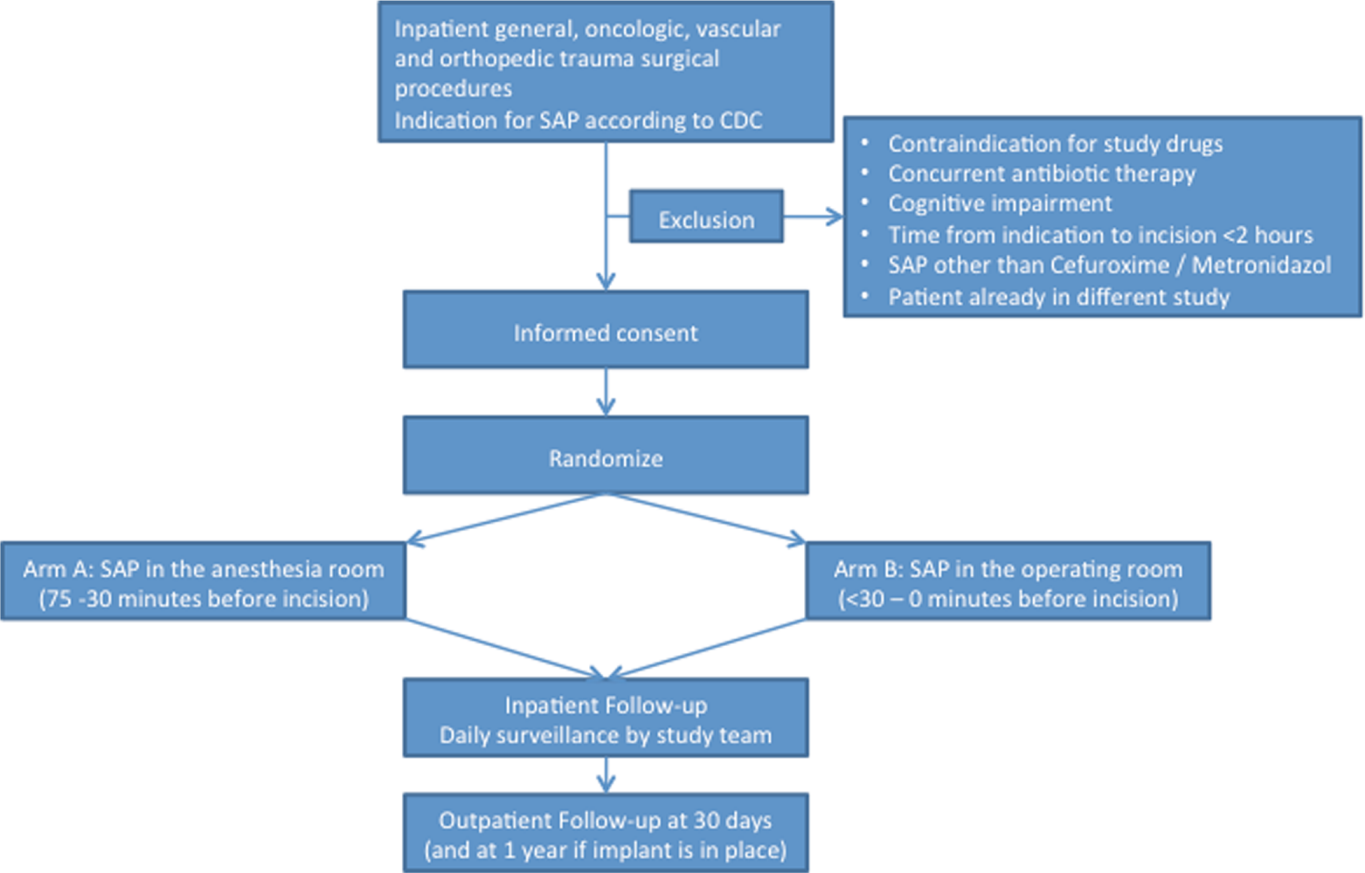
**Study flow chart.** CDC: Centers for Disease Control and Prevention; SAP: surgical antimicrobial prophylaxis.

Eligible patients are informed about the study by a member of the surgical team and receive a patient information sheet explaining the rationale and procedures of the study. The information sheet is available in Albanian, English, French, German, Italian, Portuguese, Serbo-Croatian, Spanish and Turkish. Patient information and recruitment is continuously assisted and supported by members of the study team. Written informed consent is obtained from each patient prior to randomization (Figure 1).

### Inclusion criteria

Inpatients aged 18 years or older undergoing general, oncologic, vascular and orthopedic trauma procedures with an indication for SAP according to clinical standards are eligible for this study.

Clinical standards for SAP administration are based on CDC guidelines for surgical wound classification as follows: class I (clean) involving an implant (for example, hernia mesh repair and trauma surgery), and most major vascular and breast surgery procedures; class II (clean-contaminated) procedures (for example, colorectal, small intestinal, gastroesophageal and biliary surgery); and class III (contaminated) procedures when the source of infection is surgically removed, obviating the need for xantibiotic therapy (for example, surgery for uncomplicated appendicitis, cholecystitis) [1]. SAP is not indicated for CDC class IV (dirty-contaminated) wounds. This wound class suggests that the organisms causing postoperative infection were present in the operative field before the operation, and patients are frequently receiving therapeutic antimicrobial agents perioperatively for established infections. Therefore, neither the term SAP nor the term SSI is correct in such procedures.

### Exclusion criteria

Patients are excluded on the following bases:

•Outpatient surgery

•Contraindication for study drugs, in particular penicillin type I allergy

•Pre-existing antibiotic therapy within 14 days of surgery

•Any doubt that the patient can make the decision to participate fully informed because of cognitive impairment, such as in critically ill patients or those with dementia

•Combined operations between general, oncologic, vascular or orthopedic trauma surgery with other surgical disciplines not participating in this trial

•Indication for SAP other than cefuroxime and/or metronidazole

•Patients who have already been included in other interventional studies, unless specific permission has been granted in accordance with local ethics committee guidelines

•Emergency procedures with planned incision within two hours after the surgeon indicated the procedure.

In the case of the latter criterion, emergency procedures within two hours may not allow proper patient information and randomization and we want to exclude any risk that the procedure could be delayed by participation in this study. Patients scheduled for less urgent but non-elective procedures with planned incision more than two hours after the time of indication are eligible for participation in this study since evidence for the correct timing of SAP in such procedures is needed and cannot simply be deduced from elective procedures. Thus, the results of this trial potentially have an impact on the prevention of SSI in patients undergoing such procedures in the future. However, if obtaining appropriate informed consent is jeopardized by the urgency of the procedure, the patient will be excluded and data on the reason for individual exclusion will be collected.

### Randomization and intervention

After written informed consent is obtained from eligible patients, they are randomized electronically and stratified by hospital, according to pre-existing randomization lists. Randomization results are presented both electronically and printed to the anesthesiologist responsible for SAP administration. Patients are randomized in a 1:1 ratio to have SAP administered in the anesthesia room, which is located next to the actual operating room (arm A), versus in the operating room itself (arm B). Patients in arm A receive the antibiotics between arrival in the anesthesia room and transfer to the operating room, corresponding to the time window of 75 to 30 minutes before the scheduled incision. Patients in arm B receive the antibiotics between arrival in the operating room and the time of incision, corresponding to the time window of less than 30 minutes to 0 minutes before the scheduled incision, preferably as close to the incision time as possible. SAP is administered by the anesthetic team to all patients via single-shot, intravenous infusion of 1.5 g or 3 g for patients ≥80 kg of cefuroxime in 100 ml of a 0.9% sodium chloride solution within 5 minutes and is combined with metronidazole (500 mg or 1,000 mg for patients ≥80 kg intravenous infusion, within 5 minutes) in colorectal patients, who receive no additional intraluminal antibiotics. Hence, the duration of the infusion is highly standardized. The anesthesiologist or anesthetic nurse who administers SAP records the exact time at which the infusion starts. Until 29 April 2013, corresponding to the inclusion of the first 221 patients, we instead recorded the time that the infusion ended. We had to make this amendment for feasibility reasons after consulting several opinion leaders in the field and the corresponding literature, and confirming that the standard in the US is to report when the infusion starts. In operations that last more than four hours, SAP is re-administered every four hours after the first dose according to clinical standards. In patients with impaired renal function, this re-dose will be adapted according to the creatinine clearance.

### Study endpoints

The primary endpoint of this study is the occurrence of any SSI within 30 days after surgery (within one year after implant surgery). SSI are defined as incisional (either superficial or deep) infection or organ-space infection according to CDC criteria [1]. Superficial incisional SSI involve skin and subcutaneous tissues only, common stitch mini-abscesses are excluded; deep incisional infections involve fascia and muscle; and organ-space infections involve any organ or space other than the incised layer of body wall that was opened or manipulated during surgery.

The operating surgeon, his team and ward residents, and the patients are unaware of assignments to treatment groups and are therefore blinded for treatment allocation. The operating surgeon and his team perform routine wound surveillance according to clinical standards including diagnosis and treatment of SSI. The resident in charge of the patient on the ward is responsible for registration of SSI, cross-checked by the attending surgeon in charge. In addition, inpatients are seen regularly by members of the study team. This ensures appropriate sensitivity to detect in-hospital events. Clinicians are not allowed to overrule study team members in arbitrary situations about diagnosing of SSI.

For post-discharge follow-up, trained investigators blinded for treatment allocation contact all patients 30 days after surgery by telephone. The past or present occurrence of SSI is assessed using a standardized questionnaire, and the physician who performed post-discharge clinical follow-up is identified. Whenever ongoing SSI are suspected, patients are investigated in the outpatient clinic of the two involved study centers, clinically relevant microbiological samples are cultured as needed, and the patient receives standard treatment. Whenever the telephone assessment suggests past SSI, primary care physicians are contacted and outpatient charts reviewed to gain additional information for validation of the event as described below.

In case of implant surgery, an additional monitoring assessment is performed one year after surgery, covering the mandatory follow-up period of one year as defined by CDC [1].

To ensure appropriate specificity, all cases of SSI are validated by a board certified infectious disease specialist who is blinded for the intervention, based on a comprehensive review of patient history, clinical findings, microbiology results and follow-up data.

Secondary endpoints of this study are all-cause 30-day mortality and length of hospital stay. In addition, we plan to evaluate the SSI-related economic burden in a matched case-control study nested within this RCT.

### Patient and procedure characteristics

The selection of demographics and assessment of patients’ health profiles are in accordance with the design used in our observational study [62], including all relevant patient characteristics, as well as preoperative laboratory values and factors that might influence wound healing. These involve, but are not limited to, American Society of Anesthesiologists (ASA) score, number of comorbidities on admission, smoking status, diabetes mellitus, immunosuppressive drugs, body mass index, preoperative albumin, wound class, type and duration of surgery, experience of the surgical team, surgical specialty, intraoperative core temperature, adherence to aseptic technique, and emergency procedure. The Study on the Efficacy of Nosocomial Infection Control and the National Nosocomial Infections Surveillance System (NNIS) SSI risk indices are based on some of these variables and will be calculated for each patient.

### Study management and administration

Data management and monitoring is supported by the Clinical Trial Unit of the University Hospital of Basel. Source data of every study participant are entered into an electronic data capturing system (eOPPS/ISOP; ProtecData AG, Boswil, Switzerland) and secondarily transferred into the study data management system SecuTrial® (interActive Systems GmbH, Berlin, Germany).

### Quality control measures

#### Monitoring

Continuous central and on-site monitoring of the study is performed by the Clinical Trial Unit for quality control and assurance purposes; to evaluate the progress of the study; to verify the accuracy and completeness of case report forms (CRFs) ; to ensure that all protocol requirements are met, and all applicable local authority regulations and investigator’s obligations are being fulfilled; and to resolve any inconsistency in the study records. Monitoring will consist of one initiation visit (12 hours), two monitoring visits (two days each) per year and one close-out visit (12 hours) each per center.

### Sample size considerations and statistical analyses

#### Sample size consideration

We base our sample size consideration on the planned 1:1 ratio between the two groups of patients (SAP administered 75 to 30 minutes (arm A) versus less than 30 minutes to 0 minutes (arm B) before surgery), according to the results of our observational study conducted in the years 2000 to 2001 at the University Hospital of Basel [62]. In this study, an average SSI rate of 4.7% in 3,836 general, oncologic, vascular and orthopedic trauma surgery procedures was observed. This rate varied between 4.7% and 6.8% with SAP given between 29 and 0 minutes before incision, and between 2.4% and 3.4% with SAP given 75 to 30 minutes prior to incision. The main scenario for sample size calculations was that administration of SAP 75 to 30 minutes before surgery (in the anesthesia room) will result in a 33% relative reduction of SSI risk and that SAP administration less than 30 minutes before surgery (in the operating room) will result in a SSI rate of 5%. We therefore plan to randomize 5,000 patients in a 1:1 ratio, thus resulting in two groups of 2,500 patients each. Sample size calculations were conducted using the ‘sampsi’ command of Stata Software Version 11 with a power of 80% and a two-sided type I error of 5%.

#### Statistical analyses

In order to analyze the difference in SSI occurrence between the two timing groups, logistic regression models will be used with the treatment group as the main exposure variable. The main analysis will be an intention-to-treat analysis. In secondary analyses, the logistic regression model will include the following known and suspected baseline risk factors for SSI: wound classification, ASA score, NNIS score, age, body mass index categories, presence of diabetes, smoking status, adherence to aseptic technique, and experience of the surgical team.

Additional analyses of the study will assess whether the difference of SSI risk in the two timing groups differ in specific subsets of patients: age (≥65 versus <65 years), body mass index (≥30 versus <30 kg/m^2^), diabetes (with versus without), and previous or current smoker (yes versus no). The rationale for these analyses is that we suspect the pharmacokinetics of the study drugs to be different in these subgroups. This might have an impact on the efficacy of SAP in the relevant timing categories.

Descriptive analyses will show the difference in the distribution of exact timing by randomization group and further analyses will focus on the detailed association of the exact timing of SAP with SSI risk. These latter analyses will be of observational character.

### Interim analysis

One interim analysis is planned after 2,500 patients. For this interim analysis, the outcome will be 30-day SSI risk. Decisions to stop will be taken using a fully probabilistic approach [69]. Briefly, we will calculate the predictive probability to obtain a statistically significant difference between the two arms at the end of the study. If this predictive probability is less than 5%, we will stop the trial for futility. For illustration, this will occur if the observed risk ratio in the interim analysis (SAP in anesthesia room versus SAP in operating room) is exactly 1 with 100 SSI in both study arms with 1,250 patients per arm. After 2,500 patients, this would result in an estimate of the risk ratio of 1 with a 95% confidence interval from 0.77 to 1.31. The predictive probability to obtain a statistically significant difference (*P* <0.05) between the two arms is 4.6% at the end of the study (that is, after the next 2,500 patients in both arms) and thus the trial would be stopped for futility. If the predictive probability to obtain a *P*-value of less than 4.5% at the end of the study is more (or equal) than 95%, we will stop the trial early for benefit. If not stopped early for superiority, the study continues to full length and a *P*-value of less than 4.5% is necessary to call a final result statistically significant. The overall type I error for this superiority stopping rule is 4.9% and was estimated using simulations of the scenario of identical SSI risk in two study arms of 5%.

### Ethical considerations

Participation in this trial is strictly voluntary, and patients are allowed to exit the trial at any point without explanation. All eligible patients are provided an information sheet describing the study with sufficient information for them to make an informed decision about their participation in this study. In addition, patients will be informed in detail directly by members of the study team. Patients will be excluded if receipt of adequate informed consent is jeopardized by cognitive impairment or the urgency of the procedure.

The study protocol, patients’ information sheets and informed consents and their translations were approved by the two respective local ethics committees (‘Ethikkommission beider Basel’ and ‘Kantonale Ethikkommission Kanton Aargau’). Moreover, insurance coverage of general liability has been obtained by both study centers.

Patients who decline to participate in this study are treated according to clinical standards. This includes the administration of SAP in one of the two timing categories. However, these patients will not be included and no study-specific follow-up will be performed.

### Participants’ confidentiality

The participants’ confidentiality is maintained at all times. For confidentiality reasons, CRFs must not contain any personal data of study participants. Personnel from the sponsor and regulatory authorities and members of the ethics committees are obliged to respect confidentiality and to refrain from divulging the participants’ identity or any other personal information they might be aware of. Source data in the hospital’s electronic patient information systems are secured by personal passwords and handled with respect to medical secrecy.

### Archiving and data retention

The investigator will maintain all study-related records, such as CRFs, medical records, laboratory reports, informed consent documents, safety reports, information regarding participants who discontinued, and other pertinent data. All records are to be retained by the investigator as long as required by the applicable laws and regulatory requirements (10 years). Thereafter, all data will be destroyed.

The only parameters exclusively acquired for study purposes are the result of randomization and whether the 30-day follow-up (one year if an implant is *in situ*) has been performed or not.

The study is conducted in compliance with this protocol and according to Good Clinical Practice standards as well as legal regulations.

## Discussion

There is abundant literature on SAP and its benefits. However, this is the first RCT addressing the issue of exact timing of SAP. Therefore, the aim of this RCT is to provide level I evidence for optimal SAP timing, which will eventually lead to an appropriate high-grade recommendation. The results of this RCT will potentially have an important impact on current international guidelines for this universal infection control strategy and their implementation is highly likely to reduce SSI rates all over the world.

Nowadays, SSI are considered to reflect the quality of care in a hospital, as they are potentially preventable complications directly linked to surgery. SSI are a major cause of surgical patient morbidity and mortality, and the results of this RCT may translate into a significant improvement of patient safety in the future.

Furthermore, health systems are increasingly experiencing pressure to reduce costs. An estimate of the resources that may be saved by reducing the SSI-related heavy burden on patients and healthcare providers have been presented previously [63]. Hence, this RCT has the potential to become a matter of significant interest in terms of national and international healthcare economics.

## Trial status

The trial including all respective documents was approved by both local ethics committees by July 2012. The core study team members including study nurses were recruited and trained by November 2012. Anesthesiologists who administer SAP were trained in January 2013. The initiation visit by the Clinical Trial Unit took place in February 2013. The first patient was randomized at the University Hospital Basel on 21 February 2013. As of 18 February 2014, 1,492 patients have been randomized at the University Hospital Basel.

Patient recruitment at the second study center, the Hospital Aarau, started on 7 August 2013. As of 18 February 2014, 609 patients have been randomized. In total, the study centers have randomized 2,101 patients as of 18 February 2013.

We expect to enroll the calculated sample size of 2,500 patients per intervention arm (5,000 patients total) within a two to three year time period at the two study sites. This is realistic as both hospitals together perform more than 10,000 general, oncologic, vascular and orthopedic trauma surgery procedures per year, of which about two thirds include SAP.

Post-discharge follow-up extends the study period for 30 days after the last patient undergoes surgery (one year if an implant is in place). Thorough data cleaning and validation of registered SSI by board certified infectious disease specialists occur continuously during the study period and will be completed soon thereafter.

## Abbreviations

ASA: American Society of Anesthesiologists; CDC: Centers for Disease Control and Prevention; CRF: case report form; NNIS: National Nosocomial Infections Surveillance System; RCT: randomized controlled trial; SAP: surgical antimicrobial prophylaxis; SSI: surgical site infection.

## Competing interests

The authors declare that they have no competing interests.

## Authors’ contributions

EM is responsible for the setting up and coordination of the study as well as patient recruitment and data acquisition in Basel. He drafted this manuscript. He obtained funding for this trial. He has revised this manuscript critically for important intellectual content and given final approval of this final version. He agrees to be accountable for all aspects of the work in ensuring that questions related to the accuracy or integrity of any part of the work are appropriately investigated and resolved. TZ is responsible for data acquisition and follow up and he drafted this manuscript. He has revised this manuscript critically for important intellectual content and given final approval of this final version. He agrees to be accountable for all aspects of the work in ensuring that questions related to the accuracy or integrity of any part of the work are appropriately investigated and resolved. WRM participated in the conception and design of the study. He participated in drafting this manuscript. He is responsible for the coordination of the study as well as patient recruitment in Aarau. He obtained funding for this trial. He has revised this manuscript critically for important intellectual content and given final approval of this final version. He agrees to be accountable for all aspects of the work in ensuring that questions related to the accuracy or integrity of any part of the work are appropriately investigated and resolved. MZ participated in the conception and design of the study. He participated in drafting this manuscript. He is responsible for all analysis and interpretation of data. He obtained funding for this trial. He has revised this manuscript critically for important intellectual content and given final approval of this final version. He agrees to be accountable for all aspects of the work in ensuring that questions related to the accuracy or integrity of any part of the work are appropriately investigated and resolved. HH participated in the setting up of the study in Basel. He is responsible for patient recruitment and data acquisition in Basel. He obtained funding for this trial. He has revised this manuscript critically for important intellectual content and given final approval of this final version. He agrees to be accountable for all aspects of the work in ensuring that questions related to the accuracy or integrity of any part of the work are appropriately investigated and resolved. CK is responsible for the intervention and data acquisition in Aarau. He has revised this manuscript critically for important intellectual content and given final approval of this final version. He agrees to be accountable for all aspects of the work in ensuring that questions related to the accuracy or integrity of any part of the work are appropriately investigated and resolved. CF is responsible for the validation of all SSI in Aarau. He participates in data acquisition in Aarau. He has revised this manuscript critically for important intellectual content and given final approval of this final version. He agrees to be accountable for all aspects of the work in ensuring that questions related to the accuracy or integrity of any part of the work are appropriately investigated and resolved. HM is part of the data acquisition team in Basel. She participated in the conception of this study. She participated in drafting this manuscript. She obtained funding for this trial. She has revised this manuscript critically for important intellectual content and given final approval of this final version. She agrees to be accountable for all aspects of the work in ensuring that questions related to the accuracy or integrity of any part of the work are appropriately investigated and resolved. LI is responsible for the recruitment of trauma patients and data acquisition in Basel. He has revised this manuscript critically for important intellectual content and given final approval of this final version. He agrees to be accountable for all aspects of the work in ensuring that questions related to the accuracy or integrity of any part of the work are appropriately investigated and resolved. AKL is responsible for the intervention and data acquisition in Basel. She has revised this manuscript critically for important intellectual content and given final approval of this final version. She agrees to be accountable for all aspects of the work in ensuring that questions related to the accuracy or integrity of any part of the work are appropriately investigated and resolved. CN is responsible for the recruitment of visceral patients and data acquisition in Basel. He has revised this manuscript critically for important intellectual content and given final approval of this final version. He agrees to be accountable for all aspects of the work in ensuring that questions related to the accuracy or integrity of any part of the work are appropriately investigated and resolved. UVH is responsible for the recruitment of visceral patients and data acquisition in Basel. He has revised this manuscript critically for important intellectual content and given final approval of this final version. He agrees to be accountable for all aspects of the work in ensuring that questions related to the accuracy or integrity of any part of the work are appropriately investigated and resolved. FV is part of the data acquisition team and participates in patient follow up in Basel. He has revised this manuscript critically for important intellectual content and given final approval of this final version. He agrees to be accountable for all aspects of the work in ensuring that questions related to the accuracy or integrity of any part of the work are appropriately investigated and resolved. MVS is responsible for patient recruitment and data acquisition in Aarau. He has revised this manuscript critically for important intellectual content and given final approval of this final version. He agrees to be accountable for all aspects of the work in ensuring that questions related to the accuracy or integrity of any part of the work are appropriately investigated and resolved. SR is responsible for patient recruitment and data acquisition in Aarau. He has revised this manuscript critically for important intellectual content and given final approval of this final version. He agrees to be accountable for all aspects of the work in ensuring that questions related to the accuracy or integrity of any part of the work are appropriately investigated and resolved. MK is part of the data acquisition team and participates in patient follow up in Basel. He has revised this manuscript critically for important intellectual content and given final approval of this final version. He agrees to be accountable for all aspects of the work in ensuring that questions related to the accuracy or integrity of any part of the work are appropriately investigated and resolved. AFW is responsible for the validation of all SSI and data acquisition in Basel. He has revised this manuscript critically for important intellectual content and given final approval of this final version. He agrees to be accountable for all aspects of the work in ensuring that questions related to the accuracy or integrity of any part of the work are appropriately investigated and resolved. DO participated in the conception and design of this trial and is responsible for all legal and ethical concerns of this study in Basel. He obtained funding for this trial. He has revised this manuscript critically for important intellectual content and given final approval of this final version. He agrees to be accountable for all aspects of the work in ensuring that questions related to the accuracy or integrity of any part of the work are appropriately investigated and resolved. RR participated in the design and conception of this trial and drafting this manuscript. She is responsible for trial methodology and coordination. She obtained funding for this trial. She has revised this manuscript critically for important intellectual content and given final approval of this final version. She agrees to be accountable for all aspects of the work in ensuring that questions related to the accuracy or integrity of any part of the work are appropriately investigated and resolved. WPW initiated, designed and concepted this study. He is the principal investigator and responsible for overall study coordination. He drafted this manuscript. He obtained funding for this trial. He has revised this manuscript critically for important intellectual content and given final approval of this final version. He agrees to be accountable for all aspects of the work in ensuring that questions related to the accuracy or integrity of any part of the work are appropriately investigated and resolved. All authors read and approved the final manuscript.

## Authors’ information

Our team has extensive experience in SSI prevention and control. We have published several observational and interventional studies in high-impact journals in this field [62-68]. The hypothesis to be tested in this RCT originates from the results of our prospective observational study [62], although others have published results that differ from ours.
